# Deficiency in interleukin-18 promotes differentiation of brown adipose tissue resulting in fat accumulation despite dyslipidemia

**DOI:** 10.1186/s12967-018-1684-3

**Published:** 2018-11-19

**Authors:** Kyosuke Yamanishi, Seishi Maeda, Sachi Kuwahara-Otani, Takuya Hashimoto, Kaoru Ikubo, Keiichiro Mukai, Keiji Nakasho, Naomi Gamachi, Yosif El-Darawish, Wen Li, Daisuke Okuzaki, Yuko Watanabe, Hiromichi Yamanishi, Haruki Okamura, Hisato Matsunaga

**Affiliations:** 1 Department of Neuropsychiatry, Hyogo College of Medicine, 1-1 Mukogawa, Nishinomiya, Hyogo 663-8501 Japan; 2 Department of Anatomy and Cell Biology, Hyogo College of Medicine, 1-1 Mukogawa, Nishinomiya, Hyogo 663-8501 Japan; 3 Department of Pathology, Hyogo College of Medicine, 1-1 Mukogawa, Nishinomiya, Hyogo 663-8501 Japan; 4 Laboratory of Tumor Immunology and Cell Therapy, Hyogo College of Medicine, 1-1 Mukogawa, Nishinomiya, Hyogo 663-8501 Japan; 5 Genome Information Research Center, Research Institute for Microbial Diseases, Osaka University, 3-1 Yamadaoka, Suita, 565-0871 Japan; 6 Hirakata General Hospital for Developmental Disorders, 2-1-1 Tsudahigashi, Hirakata, Osaka 573-0122 Japan

**Keywords:** Interleukin-18, Interleukin-18 knockout, Brown adipocytes, Brown adipose tissue, Lipid metabolism, Adipogenesis

## Abstract

**Background:**

The cytokine, interleukin-18 (IL-18), was originally identified as an interferon-γ-inducing proinflammatory factor; however, there is increasing evidence suggesting that it has non-immunological effects on physiological functions. We have previously investigated the potential pathophysiological relationship between IL-18 and dyslipidemia, non-alcoholic fatty liver disease and non-alcoholic steatohepatitis, which were mediated by lipid energy imbalance. Therefore, herein we focused on brown adipocytes (BAs) and brown adipose tissue (BAT) related to energy consumption as non-shivering thermogenesis.

**Methods:**

*Il18*^−/−^ male mice were generated on the C57Bl/6 background, and littermate C57Bl/6 *Il18*^+/+^ male mice were used as controls. To reveal the direct effect of IL-18, primary cell cultures derived from both mice were established. Moreover, for molecular analysis, microarray, quantitative reverse transcription PCR and western blotting were performed using 6 and 12 weeks old mice. To evaluate the short- and long-term effects of IL-18 on BAT, recombinant IL-18 was administered for 2 and 12 weeks, respectively.

**Results:**

Compared with *Il18*^+/+^ mice, BAT of *Il18*^−/−^ mice showed earlier differentiation and lipid accumulation. To examine the direct effect of IL-18 on BAT, BA cell cultures were established. *Myogenic factor 5*-expressing adipose precursor cells were extracted from *Il18*^+/+^ and *Il18*^−/−^ mice. PR domain containing 16 (PRDM16), a differentiation inducer, was strongly expressed in *Il18*^−/−^ BAs, and uncoupling protein 1, a thermogenic and differentiation marker, was upregulated, resulting in the promotion of BA differentiation. Moreover, PRDM16-dependent and independent molecules related to BAT function, such as fibroblast growth factor 21, were activated. These findings were confirmed by comparing *Il18*^+/+^ and *Il18*^−/−^ mice at 6 and 12 weeks of age. Additional analyses of the molecular mechanisms influencing the ‘Quantity of adipocytes’ identified three associated genes, apolipoprotein C3 (*Apoc3*), insulin-induced gene 1 (*Insig1*) and vitamin D (1,25-dihydroxyvitamin D3) receptor (*Vdr*). Intravenous administration of IL-18 not only significantly improved the expression of some of these genes, but it also significantly decreased the adipocytes’ size.

**Conclusions:**

This study demonstrated the critical function of IL-18 in differentiation and lipid metabolism in BAs. Furthermore, IL-18 may contribute to novel treatments by improving the energy imbalance.

**Electronic supplementary material:**

The online version of this article (10.1186/s12967-018-1684-3) contains supplementary material, which is available to authorized users.

## Background

The prevalence of obesity and dyslipidemia is currently increasing, resulting in elevated morbidity of metabolic syndromes [1]. Obesity is closely related to changes in the serum concentration of important lipid biomarkers, as well as to dyslipidemia and the development of complications such as arteriosclerosis [1, 2]. Despite the large number of patients suffering from these conditions, the factors contributing to dyslipidemia and their effective treatment remain to be elucidated.

The cytokine, interleukin-18 (IL-18), was originally identified as an interferon (IFN)-γ-inducing proinflammatory factor. However, there is increasing evidence to support it has non-immunological effects on physiological functions [3, 4]. IL-18 is produced as an inactive 24-kDa precursor and is processed by inflammasomes into an active 18-kDa mature form [5–8]. Previous studies have reported that IL-18-deficient mice developed hyperphagia, obesity and insulin resistance [9]. Moreover, these mice presented with dyslipidemia, non-alcoholic fatty liver diseases (NAFLD) and non-alcoholic steatohepatitis (NASH), and they were also related to kidney function [10, 11]. In human studies, the serum concentration of IL-18 was significantly higher in patients with type 2 diabetes mellitus and with metabolic syndrome than in healthy controls [12, 13].

Brown adipocytes (BAs) have been shown to be differentiated from myogenic factor 5 (*Myf5*)-positive progenitors, and PR domain containing 16 (PRDM16), a transcription regulator of differentiation, induced differentiation into BAs and not skeletal muscle [14]. PRDM16 acts as a transcription co-regulatory factor by activating peroxisome proliferator-activated receptor γ (PPARγ), which activates the PPARγ coactivator 1-α (*Pgc1α*) promoter [15, 16]. BAs and brown adipose tissue (BAT) express the unique molecule, uncoupling protein 1 (UCP1), which uncouples ATP production from oxidative phosphorylation, resulting in thermogenesis in mitochondria [17–20]. BAT is indispensable for basal and inducible energy generation in mammals [21, 22]. In addition, not only infant, but also adult humans have activated BAT for non-shivering thermogenesis. BAT is a very insulin-sensitive tissue, and chronic high-fat diet resulting in insulin resistance caused reduced glucose uptake by BAT in rodents, which led to diabetes [21, 23]. Decreased BAT activation has been shown in obese humans, and BAT dysfunction has been associated with obesity and metabolic diseases [24–26]. Therefore, BAT has an important role in maintaining human energy metabolism, especially the lipid balance.

A previous study has demonstrated that *IL*-*18*-knockout (*Il18*^−/−^) mice have a remarkably increased body weight accompanied by high insulin resistance and dyslipidemia [9, 10]. We have previously shown that IL-18 administration not only remarkably improved dyslipidemia, NAFLD and NASH in *Il18*^−/−^ mice, but it also inhibited body weight gain in *Il18*^+/+^ mice [10]. These results suggest there are certain relationships between IL-18 and glucose and lipid homeostasis. However, the pathophysiological mechanism and relationship between dyslipidemia and adipose tissue remain unclear.

Therefore, we investigated whether IL-18 promotes efficient use of energy through lipid metabolism in BAs and BAT. Furthermore, we examined whether IL-18 affects the differentiation and growth of BAs and BAT. To verify these hypotheses, we analyzed the role of IL-18 in the regulation of lipid concentration and cell growth in BAs and BAT as follows: (i) histopathological examination of BAs and BAT from *Il18*^+/+^ and *Il18*^−/−^ mice; (ii) analysis of primary cell cultures was performed in vitro, and thermogenic and adipogenic genes were compared; (iii) we analyzed the molecular mechanisms affected during metabolic diseases; and (iv) we assessed the lipid-normalizing effect of recombinant IL-18 (rIL-18).

## Methods

### Animals

*Il18*^−/−^ male mice were generated on the C57Bl/6 mouse background as previously described [27]. Littermate C57Bl/6 *Il18*^+/+^ male mice were used as controls. Mice were housed in groups of 3–5 in polycarbonate cages placed in a colony room maintained at a constant temperature (22 ± 1 °C) and humidity (50–60%), under a 12-h light/dark cycle (lights on at 8 a.m) with free access to standard food (MF, Oriental Yeast Co., Ltd., Tokyo, Japan) and water. All mice were sacrificed at 10 a.m. Samples from *Il18*^+/+^ and *Il18*^−/−^ mice for molecular, biochemical and histological analyses were taken at the same time points with n = 12–18, excluding in vitro and rIL-18 treatment experiments. Five to six and three mice per group were included in the short- and long-term rIL-18 treatment groups, respectively. The rIL-18 treatment details are described in the section, “Short- and long-term treatment of mice with rIL-18”.

Animal experiments were conducted according to the “Guide for Care and Use of Laboratory Animals” published by the National Institutes of Health (NIH), and were approved by the Animal Care Committee of Hyogo College of Medicine (#28041 and #14-020).

### Cell culture

The stromal vascular fraction containing adipose precursor cells was dissected from the interscapular brown fat pads of the *Il18*^+/+^ and *Il18*^−/−^ male mice at 6 weeks of age. For cell culture, tissues from 5 to 9 animals were pooled and the equivalent of 1.5 animals was used for one culture. The tissues were minced and incubated in a buffer containing 2 mg/ml collagenase Type I (Wako Pure Chemical Industries, Ltd., Osaka, Japan) and 2% bovine serum albumin for 1 h at 37 °C in a shaking bath at 100 cycles/min. Digested tissues were filtered through a 100-μm cell strainer (BD Biosciences, Tokyo, Japan) and centrifuged at 1500 rpm (440×*g*) for 10 min. The cell pellet was treated with an ammonium–chloride–potassium (ACK) solution and centrifuged again. The cells were suspended in Dulbecco’s modified Eagle’s high glucose medium supplemented with 10% fetal bovine serum and 1% penicillin/streptomycin (Nacalai Tesque, Inc., Kyoto, Japan), and seeded on 6-well plates after filtration through a 40-μm cell strainer (BD Biosciences). Then, cells were plated overnight, and attached cells were washed with phosphate-buffered saline (PBS) and grown for about 3 days in a humidified atmosphere of 5% CO_2_ and 20% oxygen until confluence. When the cells reached confluence, 0.5 mM 3-isobutyl-1-methylxanthine (IBMX) and 1 μM dexamethasone (Dex) were added to induce differentiation with or without rIL-18 (100 ng). Then, the cells were maintained in differentiation medium (10 μg/ml insulin, 50 nM triiodothyronine) for 7 days; the medium was changed every 2–3 days. The fully differentiated cells in the presence or absence of rIL-18 were harvested on day 7.

### Histological analysis

In the in vitro study, fully differentiated BAs were fixed with 4% periodate-lysine-paraformaldehyde for 1 h and processed for histological staining with Oil Red O.

In the in vivo experiment, three to five mice per group were used for histopathological analysis. Mice were deeply anesthetized with isoflurane and perfused transcardially with periodate-lysine-paraformaldehyde fixative at 10 a.m. The fixed BATs were removed, cut into small pieces and immerged in the same fixative at 4 °C overnight. The specimens were processed for histological staining by embedding sections in paraffin for hematoxylin and eosin staining. Stained sections were mounted, and pathological diagnosis was determined in a blinded fashion by specialists. Stained cells and tissue sections were photographed using an optical microscope and CCD camera (AX-80 and DP-71, Olympus, Tokyo, Japan).

### Molecular analysis

The protocols for sample collection, mRNA purification, microarray, Ingenuity^®^ Pathway Analysis (IPA; Ingenuity^®^ Systems, Redwood, CA, USA) and quantitative reverse transcription PCR (qRT-PCR) have been described previously [10, 11, 28]. Mice were euthanized by decapitation at 10 a.m, and BATs were removed and immediately placed in liquid nitrogen, and then kept at − 80 °C until use.

Total RNA was purified from mouse BAT using a Sepasol-RNA I Super kit (Nacalai Tesque, Kyoto, Japan) according to the manufacturer’s instructions, and treated with 5 units of RNase-free DNase I at 37 °C for 30 min to remove genomic DNA contamination. After phenol/chloroform extraction and ethanol precipitation, total RNA was dissolved in deionized distilled water. RNA concentrations were determined by spectrophotometry.

For microarray analysis, expression profiling was performed using SurePrint G3 Mouse GE 8 × 60 K Microarray G4852A (Agilent Technologies Inc., Santa Clara, CA, USA). Twelve microarray analyses as a one-color experiment were performed in biological triplicates. Each gene expression profile was compared between *Il18*^+/+^ and *Il18*^−/−^ mice at 6 and 12 weeks of age. Total RNA (200 ng) was reverse transcribed into double-stranded cDNA using AffinityScript multiple temperature reverse transcriptase (Agilent Technologies Inc.), and amplified. The resulting cDNAs were used for in vitro transcription by T7-polymerase and labeled with cyanine-3-labeled cytosine triphosphate (Perkin Elmer, Wellesley, MA, USA) using a Low Input Quick-Amp Labeling Kit (Agilent Technologies Inc.). After the labeled cDNAs were fragmented, each cDNA sample was hybridized to SurePrint G3 Mouse GE 8 × 60 K Microarray (Agilent Design # 028005). After washing, the slides were scanned with an Agilent Microarray Scanner (G2505C). Feature extraction software (version 10.5.1.1; Agilent Technologies Inc.) was used to convert images into gene expression data. For microarray data analysis, raw data were imported into Subio platform version 1.18 (Subio Inc., Kagoshima, Japan), and raw intensity data were normalized to the 75th percentile intensity of probes above the background levels (gIsWellAbove = 1). The BAT genes in *Il18*^+/+^ and *Il18*^−/−^ mice were defined to show signal ratios of > 2.0-fold and < 0.5-fold. Details of the microarray analysis and results can also be found in the Gene Expression Omnibus (GEO; Accession No. GSE64308).

Heatmaps were generated using the open source web tool, ClustVis (https://biit.cs.ut.ee/clustvis/), as previously described [29].

IPA software was used for microarray analysis for the interpretation of gene expression data. The IPA network explorer was used to detect relevant interactions among the *Il18*^+/+^ and *Il18*^−/−^ genes, and identify the shortest direct paths between genes [10, 28].

For qRT-PCR, BAT samples at 6 and 12 weeks of age were obtained from the same animals used for the microarray analysis. Total RNA (10 ng/reaction) was used in the RNA-direct SYBR Green Real-Time PCR Master Mix: One-step qPCR kit (Toyobo Co. Ltd., Tokyo, Japan). Samples were run in duplicate reactions in 96-well plates. Median threshold cycle values were used to calculate the fold changes (FC) between the samples of the groups. The FC values were normalized to glyceraldehyde 3-phosphate dehydrogenase (*Gapdh*) or β-actin (*Actb*) levels. The following temperature profile was used according to the manufacturer’s instructions: 30 s at 90 °C and 20 min at 61 °C for reverse transcription, followed by 45 cycles of 98 °C for 1 s, 67 °C for 15 s and 74 °C for 35 s. The primer sequences for qRT-PCR are shown in Additional file 1.

### Western blotting

Mouse BAs/BATs were minced in lysis buffer (1% Nonidat P-40, 20 mM Tris–HCl, pH 8.0, 150 mM NaCl, 10% glycerol) containing protease inhibitor cocktail (Complete™, Roche, Mannheim, Germany), and then homogenized on ice using a sonicator (Sonifier II, Branson, MO, USA). Each lysate was centrifuged at 13,000×*g* for 3 min and the supernatant was collected. The protein concentration of each specimen was determined with a Bradford protein assay kit (Bio-Rad Laboratories, Hercules, CA, USA). Samples were denatured in Laemmli sample buffer for 5 min at 95 °C, electrophoresed in a 12.5% sodium dodecyl sulfate polyacrylamide gel, and transferred onto polyvinylidene difluoride membranes (Hybond-P, Amersham Bioscience, Little Chalfont, UK). The antibodies used were monoclonal rabbit anti-β-actin (ACTB; Cat. No. 5125S; Cell Signaling Technology, Inc., Danvers, MA, USA), adipose triglyceride lipase (ATGL; Cat. No. 2439, Cell Signaling), fatty acid binding protein (FABP4; Cat. No. 3544, Cell Signaling), fibroblast growth factor 21 (FGF21; Cat. No. LS-B5864, LifeSpan Biosciences, Inc. Seattle, WA, USA), IL-18 (IL18; Cat. No. D046-3, Medical & Biological Laboratories Co., Ltd., Tokyo, Japan), monoclonal rabbit anti-GAPDH (Cat. No. 3683S; Cell Signaling Technology, Inc.), PGC1α (Cat. No. Ab3242, Merck Millipore, MA, USA), Ser563-phosphorylated hormone-sensitive lipase (pHSL563; Cat. No. 4139, Cell Signaling), Ser565-phosphorylated HSL (pHSL565; Cat. No. 4137, Cell Signaling), PPARγ (Cat. No. 2430, Cell Signaling), PRDM16 (Cat. No. Ab106410, Abcam plc, Cambridge, UK) and UCP1 (Cat. No. Ab10983, Abcam plc). Membranes were blocked with 1% bovine serum albumin in PBS containing 0.1% Triton X-100 (TPBS), incubated with primary antibodies at 4 °C overnight, followed by incubation with horseradish peroxidase-conjugated secondary antibodies (#NA9340V and #RPN1025, GE Healthcare, Buckinghamshire HP7 9NA, UK). Washing with TPBS was performed after each treatment. Antibody reactions were captured using the photo-image analyzer, LAS-4010 (Fuji Photo Film Co., Ltd., Tokyo, Japan). The density of specific protein bands was measured with ImageJ (http://rsbweb.nih.gov/ij/, version 1.6), and the results obtained were normalized to β-actin levels. The mean of measured bands in the controls was set to one. We also assessed positive controls.

### Short- and long-term treatment of mice with rIL-18

To determine the response to rIL-18 treatment, mice were administered 2 µg of rIL-18 dissolved in saline containing heat-inactivated normal mouse serum (0.5%). They were injected twice a week via the caudal vein for 2 weeks (short-term study) from 10 weeks of age, and for 12 weeks (long-term study) from 12 or 37 weeks of age, as previously reported [10]. For control experiments, saline was injected using the same procedure. Five to six and three mice per group were included in the short- and long-term treatment groups, respectively.

### Statistical analysis

All results are expressed as mean ± SD. Sigmaplot™ (version 11.0 Systat Software, Inc., San Jose, CA, USA) was used for all statistical analyses. Body weight, serum measurement, qRT-PCR and western blotting were analyzed by the Student *t*-test or Mann–Whitney *U*-test. Analysis of rIL-18 administration was examined by two-way analysis of variance (ANOVA). Correlations between microarray and qRT-PCR were examined by Spearman’s rank correlations test. Differences were considered statistically significant when *p* < 0.05. All analyses were performed at least twice to confirm the results.

## Results

### The differentiation ability of the BA progenitors

First, we confirmed there were no direct or indirect interactions between IL-18 and molecules related to cell differentiation and thermogenesis in BAs using the IPA database (Additional file 2).

**Fig. 1:**
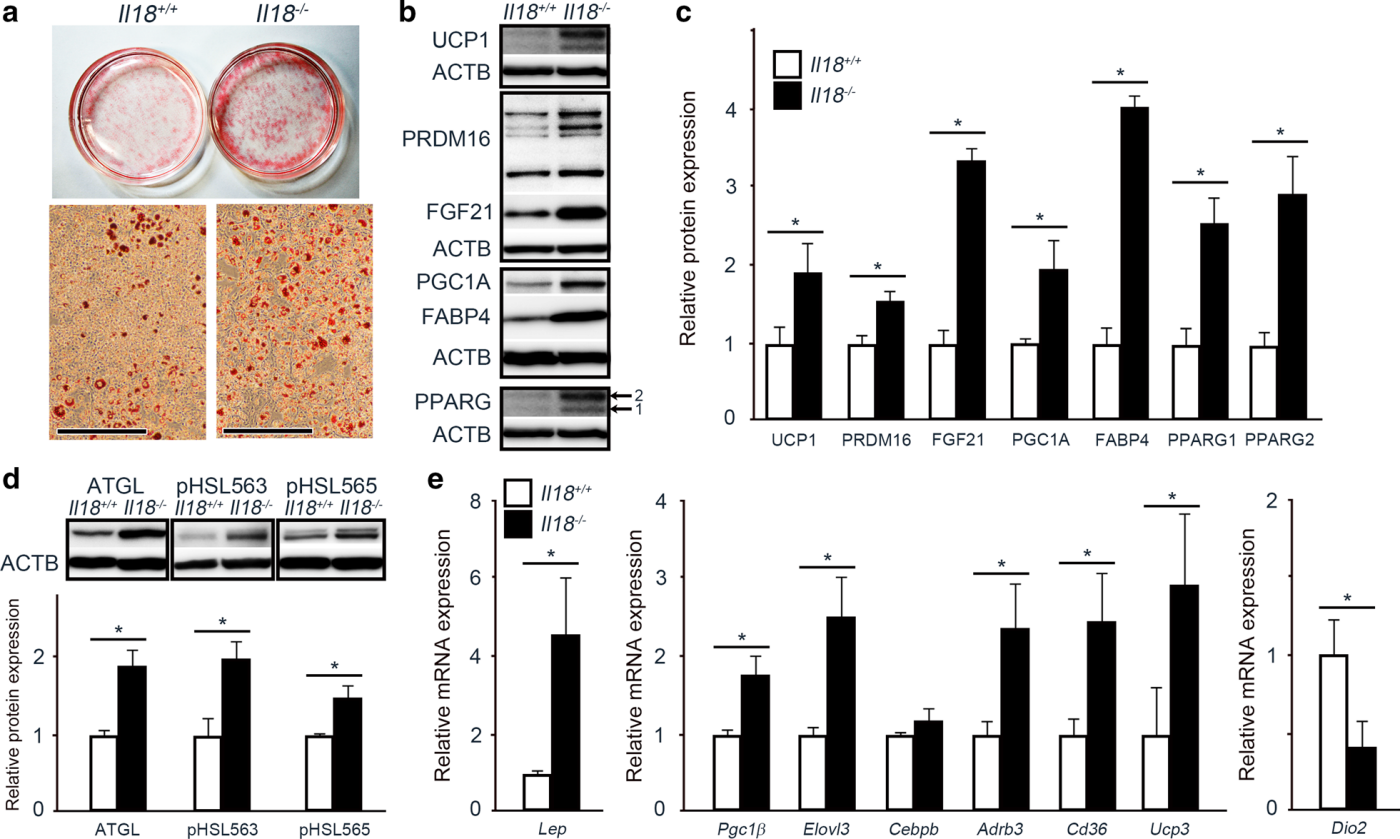
Brown adipose precursor cells from *Il18*^−/−^ mice differentiate much more than cells from *Il18*^+/+^ mice. **a** Histopathological analysis of differentiated BAs with Oil Red O staining. **b**, **c** The protein expression of differentiation and thermogenic markers in each group was compared. **d** The expression of lipolysis markers. **e** The expression of other molecules related to BA function in *Il18*^−/−^ and *Il18*^+/+^ mice. Data are presented as mean ± SD (**b**–**e**; n = 3 per group), *p < 0.05. All experiments were repeated at least three times. Scale bars, 50 μm (**a**). ACTB: β-actin; *Adrb3*: adrenergic receptor β 3; ATGL: adipose triglyceride lipase; *Cd36*: cluster of differentiation 36; *Cebpb*: CCAAT/enhancer binding protein (C/EBP) beta; *Dio2*: type II iodothyronine deiodinase; *Elovl3*: elongation of very long chain fatty acids protein 3; FABP4: fatty acid-binding protein 4; FGF21: fibroblast growth factor 21; *Lep*: leptin; PGC1α: peroxisome proliferator-activated receptor γ coactivator 1-α; *Pgc1b*: peroxisome proliferator-activated receptor γ coactivator 1-β; pHSL563: phospho-HSL (Ser563); pHSL565: phospho-HSL (Ser565); PPARγ: peroxisome proliferator-activated receptor γ; PRDM16: PR domain containing 16; UCP1: uncoupling protein 1; *Ucp3*: uncoupling protein 3

The differentiation of BAs after induction is shown in Fig. 1a. BAs from *Il18*^−/−^ mice were more differentiated and contained more lipids than those from *Il18*^+/+^ mice (Fig. 1a). Furthermore, when we examined the protein expression of the induction marker, PRDM16, it was significantly higher in cells from *Il18*^−/−^ mice than in cells from *Il18*^+/+^ mice (Fig. 1b, c). In addition, the markers, UCP1, FGF21, PGC1α, FABP4, PPARγ1 and PPARγ2, showed significantly increased expression in cells from *Il18*^−/−^ mice compared with that in *Il18*^+/+^ mice (Fig. 1b, c). The expression of the lipolysis markers, ATGL and pHSL, was higher in cells from *Il18*^−/−^ mice compared with cells from *Il18*^+/+^ mice (Fig. 1d). Moreover, the mRNA expression of *Lep*, *Pgc1b*, *Elovl3*, *Adrb3*, *Cd36* and *Ucp3* was higher in cells from *Il18*^−/−^ mice compared with cells from *Il18*^+/+^ mice, whereas *Cebpb* was unchanged, and *Dio2* was significantly suppressed (Fig. 1e).

### Histopathological observations of BAT during growth in vivo

**Fig. 2:**
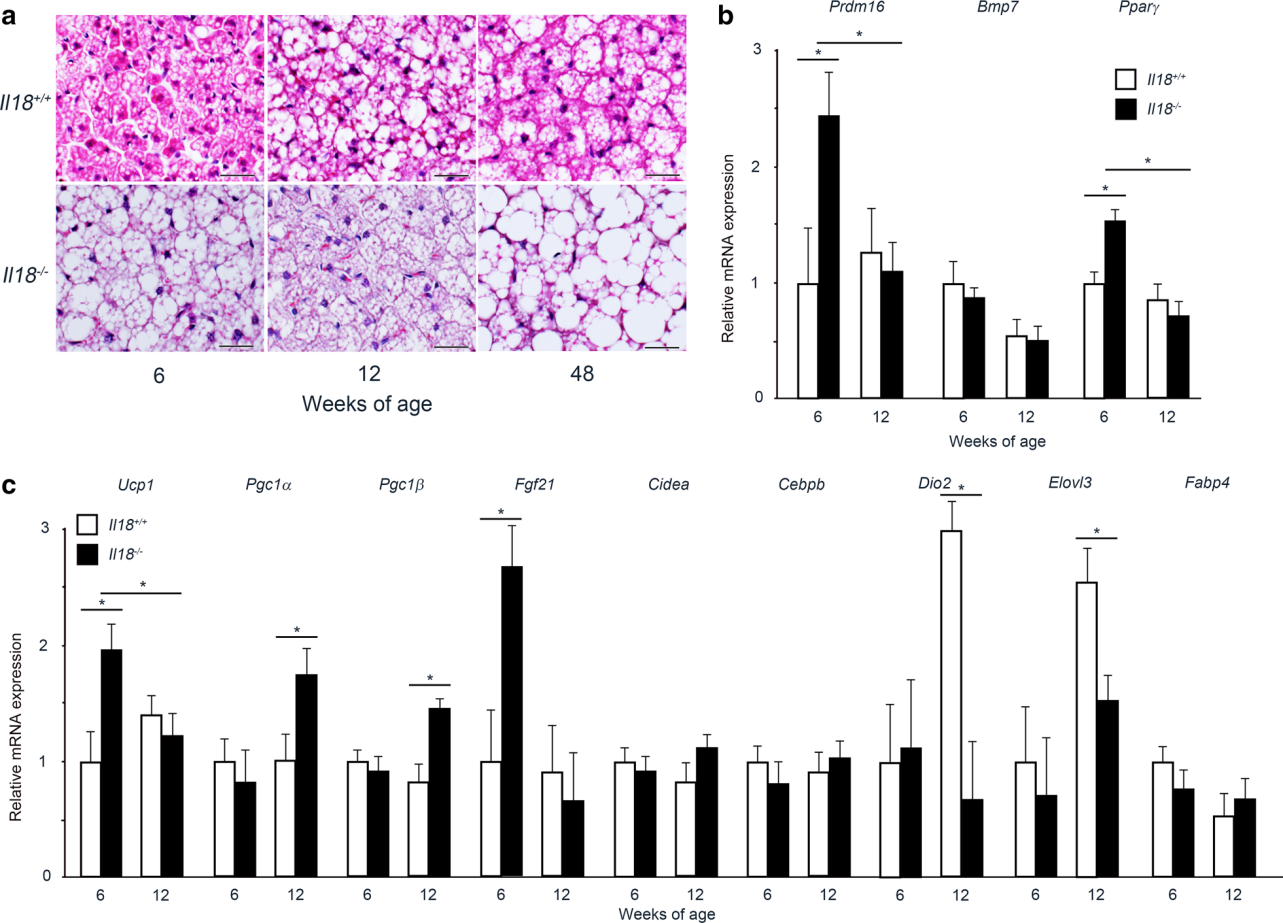
BAT in *Il18*^−/−^ mice spontaneously accumulates fat. **a** To analyze lipid accumulation in BAT from *Il18*^−/−^ mice during growth, histopathology was performed at 6, 12 and 48 weeks of age by staining with hematoxylin and eosin (**a**). Scale bars, 50 μm. **b**, **c** The mRNA expression of molecules related to differentiation and thermogenesis in BAT at 6 and 12 weeks of age. Data are presented as mean ± SD (n = 4–6 mice per group), *p < 0.05. *Bmp7*: bone morphogenetic protein 7; *Cebpb*: CCAAT/enhancer binding protein (C/EBP) β; *Cidea*: cell death activator CIDE-A; *Dio2*: type II iodothyronine deiodinase; *Elovl3*: elongation of very long chain fatty acids protein 3; *Fabp4*: fatty acid-binding protein 4; *Fgf21*: fibroblast growth factor 21; *Pgc1a*: peroxisome proliferator-activated receptor γ coactivator 1-α; *Pgc1b*: peroxisome proliferator-activated receptor γ coactivator 1-β; *Pparg*: peroxisome proliferator-activated receptor γ; *Prdm16*: PR domain containing 16; *Ucp1*: uncoupling protein 1

Histopathological appearances were examined in BAT of *Il18*^−/−^ and *Il18*^+/+^ mice at 6, 12 and 48 weeks of age. At 6 weeks of age, more lipids were observed in BAT from *Il18*^−/−^ mice than in that from *Il18*^+/+^ mice (Fig. 2a). At 12 weeks of age, a clear difference was not observed. However, at 48 weeks of age, significantly more and larger lipid droplets, and squamous nuclei were observed in *Il18*^−/−^ mice than in *Il18*^+/+^ mice (Fig. 2a).

### Comparison of BA differentiation and lipid metabolism-related molecules in vivo

To confirm the mRNA expression profile differences observed in vitro, qRT-PCR was performed. First, molecules related to BA differentiation were compared. At 6 weeks of age, *Prdm16* and *Pparg* expression in *Il18*^−/−^ mice was significantly higher than that in *Il18*^+/+^ mice, though no difference was observed at 12 weeks of age (Fig. 2b). Next, the expression of thermogenic and adipogenic genes in each group was analyzed. At 6 weeks of age, *Ucp1* and *Fgf21* in *Il18*^−/−^ mice were upregulated compared with *Il18*^+/+^ mice. However, at 12 weeks of age, *Pgc1a* and *Pgc1b* were upregulated, whereas *Dio2* and *Elovl3* were downregulated (Fig. 2c). *Cidea*, *Cebpb* and *Fabp4* were similar between the groups (Fig. 2c).

### Microarray and Ingenuity^®^ Pathway Analysis

**Fig. 3:**
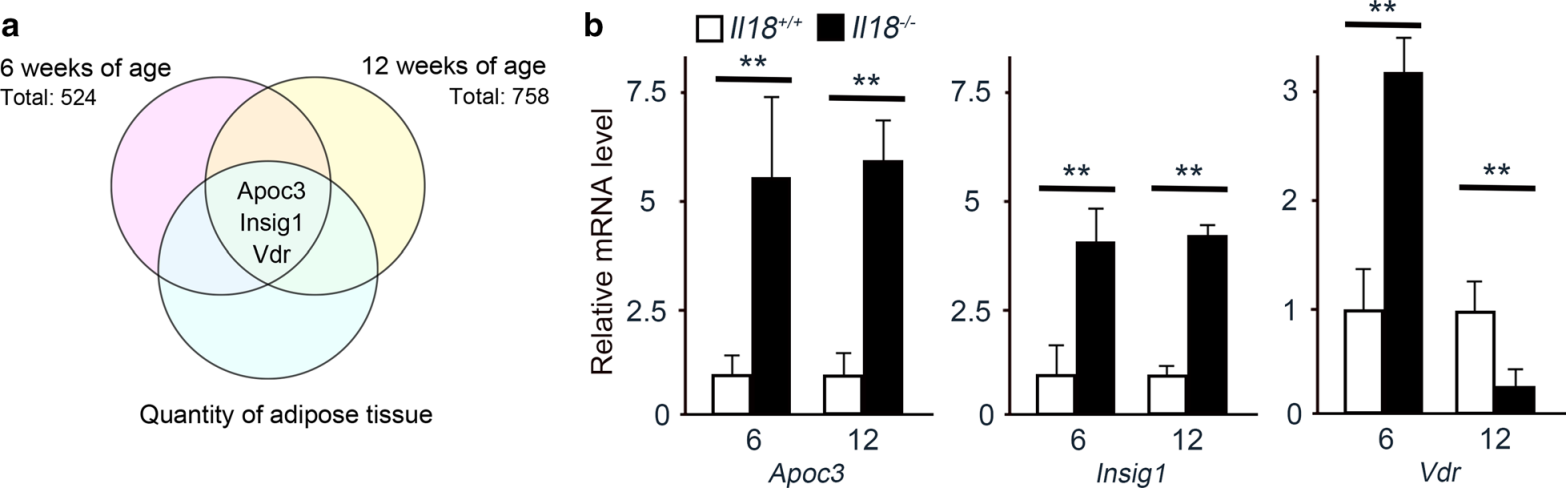
Identification of effector molecules of ‘Quantity of adipose tissue’ in *Il18*^−/−^ mice. **a** Summary of the microarray analyses of *Il18*^−/−^ and *Il18*^+/+^ BAT at 6 and 12 weeks of age. Genes with expression > 2-fold or < 0.5-fold compared with that of the *Il18*^+/+^ mice were chosen. Three molecules involved in ‘Quantity of adipose tissue’ at both 6 and 12 weeks of age are shown. **b** The mRNA expression of these molecules by microarray and qRT-PCR at 6 and 12 weeks of age (n = 3 per group). **p < 0.01. *Apoc3*: apolipoprotein C3; *Insig1*: insulin-induced gene 1; *Vdr*: vitamin D receptor

The heatmap of the microarray results at 6 and 12 weeks of age is shown in Additional file 3a and b, respectively. Among the microarray genes, molecules related to ‘Quantity of adipose tissue’ extracted from the IPA database were initially analyzed. The IPA results indicated that three molecules coding for *Apoc3*, *Insig1* and *Vdr* were involved in ‘Quantity of adipose tissue’ between 6 and 12 weeks of age (Fig. 3a). The heatmap of these three molecules at 6 and 12 weeks of age is shown in Additional file 3c and d, respectively. Additionally, all isolated genes at 6 and 12 weeks of age were categorized automatically by IPA (Additional files 4 and 5). To confirm the microarray results, qRT-PCR was performed. First, we compared the microarray and qRT-PCR results, to determine significant correlations by Spearman’s rank correlations test, which revealed a significant correlation (at 6 weeks of age: rs = 0.991, *p* < 0.001; at 12 weeks of age: rs = 0.955, *p* < 0.001). The *Apoc3* and *Insig1* expression in *Il18*^−/−^ mice at 6 and 12 weeks of age was higher than that in *Il18*^+/+^ mice (Fig. 3b). *Vdr* expression in *Il18*^−/−^ mice at 6 weeks of age was increased compared with that in *Il18*^+/+^ mice, but it was decreased at 12 weeks of age (Fig. 3b).

### Effect of rIL-18 treatment on molecules in BA progenitors in vitro

To analyze the effect of IL-18 on BA progenitors from *Il18*^−*/*−^ mice, saline or rIL-18 was added to these cells before differentiation. Surprisingly, there was no effect on molecules that were significantly different such as UCP1 in Fig. 1 (Additional file 6). In addition, we examined IL-18 expression in BA progenitor and differentiated cells from both *Il18*^+*/*+^ and *Il18*^−/−^ mice. We found no expression of IL-18 in rIL-18-treated differentiated cells from *Il18*^−/−^ mice (Additional file 7).

### The influence of short-term rIL-18 treatment on thermogenic and adipogenic molecules

**Fig. 4:**
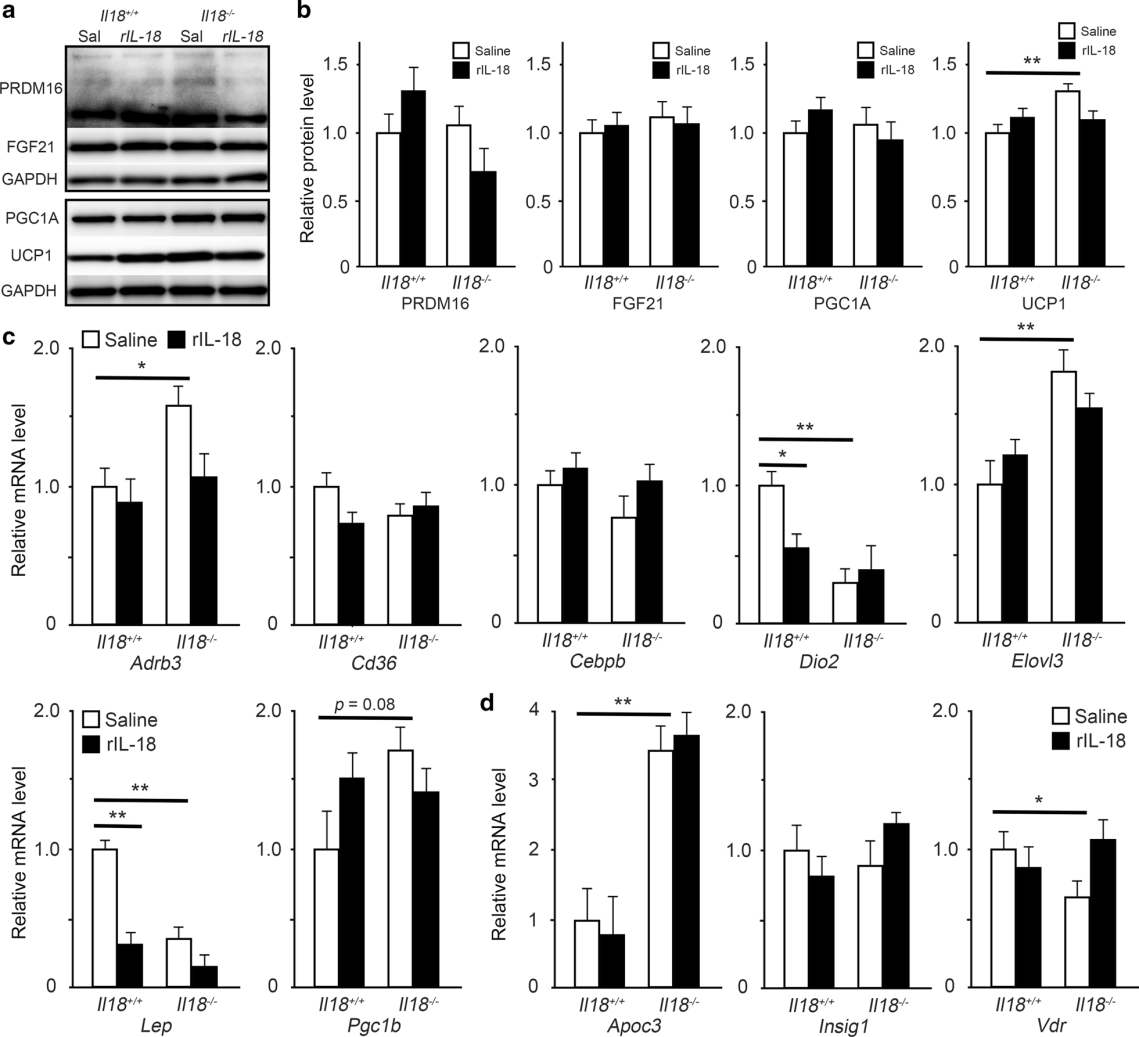
Short-term treatment with rIL-18 regulates the expression of thermogenic and ‘Quantity of adipose tissue’-related molecules. **a**, **b** Mice were injected with rIL-18 twice a week for 2 weeks from 10 weeks of age for short-term treatment. The protein expression of PRDM16, FGF21, PGC1α and UCP1 was measured. **c** The mRNA expression of the thermogenic genes, *Adrb3*, *Cd36*, *Cebpb*, *Dio2*, *Elovl3*, *Lep* and *Pgc1b* was compared between the groups. **d** The influence on molecules related to ‘Quantity of adipose tissue’ from the microarray was analyzed. (**b**–**d**, n = 4–5 per group) *p < 0.05, **p < 0.01. ACTB: β-actin; *Apoc3*: apolipoprotein C3; *Adrb3*: adrenergic receptor β 3; *Cd36*: cluster of differentiation 36; *Cebpb*: CCAAT/enhancer binding protein (C/EBP) β; *Dio2*: type II iodothyronine deiodinase; *Elovl3*: elongation of very long chain fatty acids protein 3; FGF21: fibroblast growth factor 21; *Insig1*: insulin-induced gene 1; *Lep*: leptin; PGC1α: peroxisome proliferator-activated receptor γ coactivator 1-α; *Pgc1b*: peroxisome proliferator-activated receptor γ coactivator 1-β; PRDM16: PR domain containing 16; UCP1: uncoupling protein 1; *Vdr*: vitamin D receptor

To confirm the role of IL-18 in lipid synthesis in BAT, *Il18*^+/+^ and *Il18*^−/−^ mice were treated with rIL-18. First, the expression of PRDM16, FGF21, PGC1α and UCP1 was compared. At 12 weeks of age, PRDM16, FGF21 and PGC1α were not affected by rIL-18 (Fig. 4a, b). In contrast, UCP1 expression was significantly different between the saline groups. However, the higher UCP1 expression in the *Il18*^−/−^ mice was reduced to control levels by the 2-week intravascular administration of rIL-18 (Fig. 4a, b).

The expression of *Adrb3*, *Cd36*, *Cebpb*, *Dio2*, *Elovl3*, *Lep* and *Pgc1b* was measured after 2 weeks of rIL-18 or saline administration (Fig. 4c). While the expression of *Adrb3*, *Dio2* and *Elovl3* in *Il18*^−/−^ mice that received saline was similar to that in Fig. 1e, the expression of *Adrb3* and *Elovl3* in *Il18*^−/−^ mice administered with rIL-18 was clearly reduced (Fig. 4c). In addition, *Lep* significantly decreased in both *Il18*^+/+^ and *Il18*^−/−^ mice administered with rIL-18, which was opposite to the in vitro result (Figs. 1e and 4c).

### Effect of short-term rIL-18 treatment on molecules identified by microarray

The expression of *Apoc3*, *Insig1* and *Vdr* was measured after 2 weeks of saline or rIL-18 administration (Fig. 4d). In *Il18*^−*/*−^ mice, only *Vdr* clearly responded to rIL-18 administration. *Apoc3* expression was similar to that in Fig. 3b, and it was not influenced by rIL-18 injection in both *Il18*^+*/*+^ and *Il18*^−/−^ mice (Fig. 4d).

### Effects of long-term rIL-18 administration on BAT in Il18^+/+^ mice

**Fig. 5:**
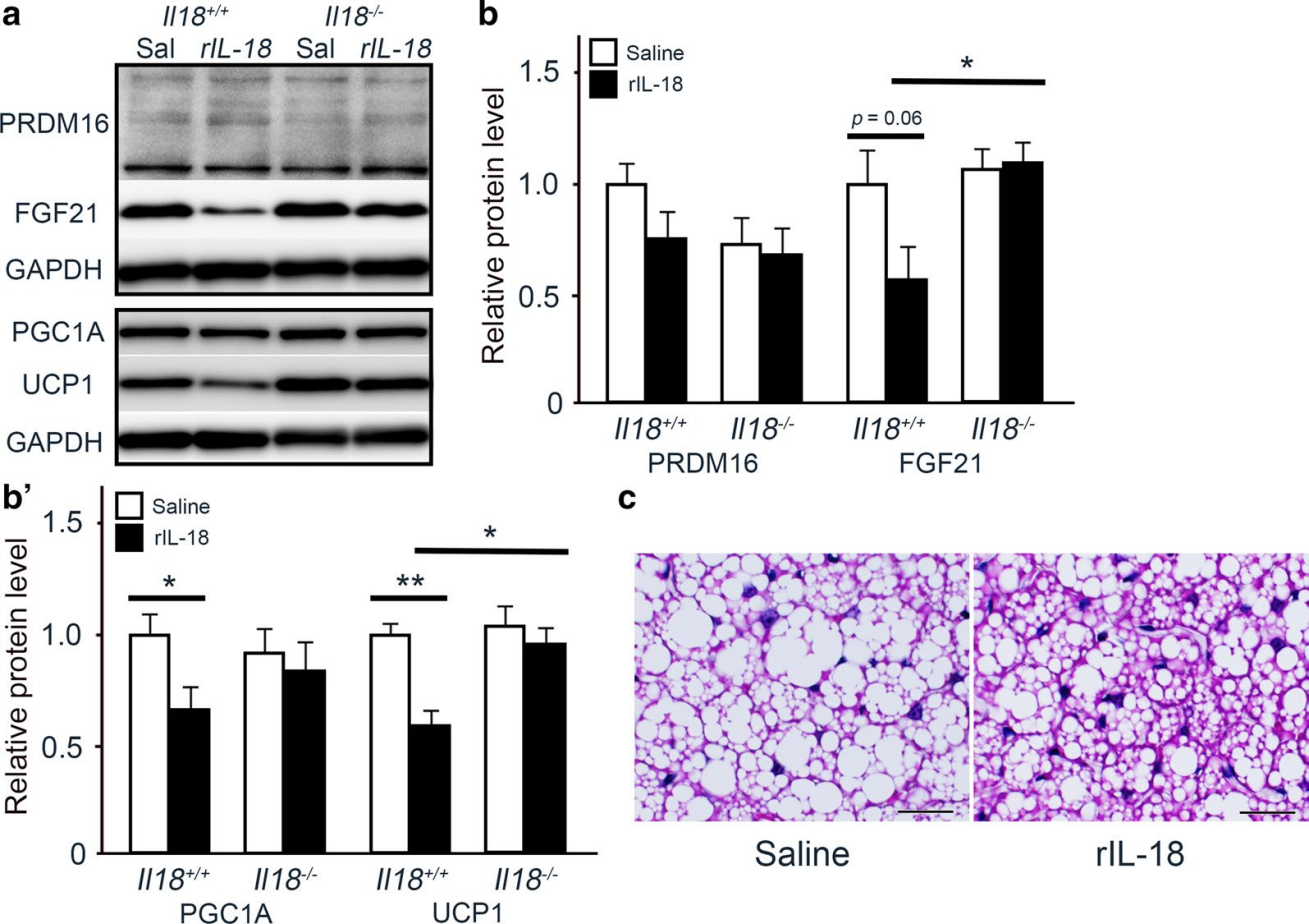
Effect of long-term rIL-18 administration on differentiation, thermogenic-related molecules and the development of lipid accumulation. **a**, **b** *Il18*^+/+^ and *Il18*^−/−^ mice were treated with rIL-18 twice a week for 12 weeks for long-term treatment from 12 weeks of age, and the protein expression of FGF21, PGC1α, PRDM16 and UCP1 was compared between the groups. **c** *Il18*^−/−^ mice were treated with rIL-18 twice a week for 12 weeks for long-term treatment from 37 weeks of age, and hematoxylin and eosin staining was performed. Scale bars, 50 μm (**b**, **c**). (**b**, n = 3 per group) *p < 0.05. FGF21: fibroblast growth factor 21; PGC1α: peroxisome proliferator-activated receptor γ coactivator 1-α; PRDM16: PR domain containing 16; UCP1: uncoupling protein 1

We have previously found that compared with mice that received saline administration, long-term administration of rIL-18 effectively inhibited body weight increase in *Il18*^+/+^ mice, but not in *Il18*^−/−^ mice [10]. Therefore, we speculated that long-term administration of rIL-18 would have some effect on adipocytes. Compared with mice that received saline administration, long-term administration of rIL-18 effectively decreased the expression of PGC1α and UCP1 in *Il18*^+/+^ mice, but not in *Il18*^−/−^ mice (Fig. 5a, b).

### Long-term rIL-18 treatment ameliorates the development of steatosis in BAT of Il18^−/−^ mice

We have also previously demonstrated that administration of rIL-18 improved the development of NAFLD and NASH [10]. BAT was similarly affected by this treatment (Fig. 5c). BAT from *Il18*^−/−^ mice treated with saline was similar to that shown in Fig. 2a. BAT from *Il18*^−/−^ mice treated with rIL-18 showed a smaller size and number of fat droplets compared with that from the same mice treated with saline (Fig. 5c).

## Discussion

In the present study we revealed the following novel pathophysiological features of IL-18 functions: (1) BAs extracted from *Il18*^−/−^ mice were more differentiated than those from *Il18*^+/+^ mice; (2) this phenomenon was induced by upregulation of the differentiation inducer, PRDM16, and resulted in not only upregulated PRDM16-dependent genes such as *Pparg* and *Pgc1a*, but also in upregulated PRDM16-independent genes such as *Fgf21*; (3) BAT in *Il18*^−/−^ mice developed severe adiposity during growth compared with that in *Il18*^+/+^ mice; (4) several molecules related to adiposity were affected by IL-18 deficiency; and (5) some of these abnormal conditions were recovered by rIL-18 administration, which also affected some of the identified molecules.

Regarding BA differentiation, in *Myf5*-positive progenitors, PRDM16 led not only to skeletal muscle differentiation, but also to BA differentiation of brown preadipocytes. Furthermore, PPARγ was indispensable for BA differentiation [14, 30, 31]. PGC1α, a coactivator of PPARγ, is a crucial regulator of mitochondrial biogenesis and oxidative metabolism, and it activates thermogenic functions [32]. This heat production depends on UCP1 activity [33]. Moreover, PRDM16 increases the transcriptional activity of PGC1α and PPARγ [34]. FGF21, a member of the FGF superfamily, is mainly released from the liver and has recently emerged as a novel regulator of glucose and lipid metabolism [35, 36]. FGF21 has been shown to promote thermogenic activity in neonatal BAT and in isolated BAs [37]. Recently, a study has demonstrated that FGF21 was secreted from BAT and that BAT has an endocrine role during thermogenic activation [38].

According to our results, BAs from *Il18*^−/−^ mice were more differentiated than those from *Il18*^+/+^ mice (Fig. 1a). The expression of PRDM16, PGC1α, PPARγ and UCP1 in *Il18*^−/−^ mice was significantly higher than that in *Il18*^+/+^ mice (Fig. 1b, c). Additionally, the relative expression of FGF21, which is a thermogenic activator in BAT, in *Il18*^−/−^ mice was significantly higher than that in *Il18*^+/+^ mice (Fig. 1b, c). These results suggest that IL-18 deficiency directly induces transcription regulators, resulting in promotion of BA differentiation, thermogenesis activation and increased energy production.

Similar to the differentiation and thermogenic regulators, FABP4 expression in BAs extracted from *Il18*^−/−^ mice was increased compared with that in BAs from *Il18*^+/+^ mice (Fig. 1b, c). FABP4 is expressed in adipose tissue, and is related to obesity and diabetes mellitus [39]. Moreover, high expression of FABP4 in adipose tissue has been suggested to increase insulin resistance [40, 41]. FABP4 is induced by PPARγ, and these two molecules interact with each other and have opposite effects on energy metabolism [42, 43]. Furthermore, in vivo, the amount of food and energy intake of *Il18*^−/−^ mice increased compared with the controls, which led to obesity, increased insulin resistance and diabetes mellitus [9]. We have also previously found that *Il18*^−/−^ mice developed dyslipidemia, NAFLD and NASH [10]. Our results suggest that higher expression of FABP4 in BAs is one of the key factors in these metabolic disorders.

ATGL and HSL in adipose tissue are key enzymes involved in intracellular degradation of triacylglycerol. Overexpression of ATGL in adipose tissue increased thermogenesis, resulting in higher energy expenditure and resistance to obesity [44]. Moreover, ablation of ATGL in BAT led to transformation of BAT into white adipose tissue (WAT)-like tissues and deceased the expression of BAT-related molecules, resulting in suppressed thermogenesis [45]. HSL is activated by phosphorylation at Ser563, leading to lipolysis [46, 47]. However, HSL phosphorylation at Ser565 suppresses HSL phosphorylation at Ser563 and inhibits HSL activity [47, 48]. In our study, the expression of ATGL, pHSL563 and pHSL565 in cells from *Il18*^−/−^ mice was significantly increased compared with that in cells from *Il18*^+/+^ mice (Fig. 1d). These results suggest that lipolysis in BAs from *Il18*^−/−^ mice was more robustly promoted than in BAs from *Il18*^+/+^ mice through HSL activation.

Further examination of other molecules related to BA differentiation and thermogenesis such as *Elovl3* and *Adrb3* was performed by qRT-PCR. *Elovl3* is a specific marker of BAT, CD36 is indispensable for thermogenesis under low temperatures, and thermogenesis is promoted by *Adrb3* signaling [49]. *Dio2* is a prohormone that converts tyrosine into triiodothyronine (T3) [50]. Signal transducer and activator of transcription 3 (STAT3) is phosphorylated by Leptin [51]. The expression of all of these molecules, except for *Dio2*, was increased under IL-18 deficiency, whereas *Dio2* was decreased (Fig. 1e). These results suggest that differentiation and thermogenesis in BAs from *Il18*^−/−^ mice were accelerated though decreased expression of *Dio2*, which may stem from a negative feedback due to T3-induced differentiation.

In vivo, while the amount of food and energy intake of *Il18*^−/−^ mice was higher compared with the controls, resulting in obesity, insulin resistance and diabetes mellitus, the metabolic rate of *Il18*^−/−^ mice was similar to that of the controls [9]. In addition, our previous study has revealed that *Il18*^−/−^ mice showed dyslipidemia, resulting in NAFLD and steatohepatitis [10]. Especially, hypertriglyceridemia occurred at 6 weeks of age, which was the same age at which we extracted adipose precursor cells for primary cell culture. Our results showed that in *Il18*^−/−^ mice, BAT stored more lipids with age (Fig. 2a). Moreover, the gene expression of *Prdm16*, *Ucp1*, *Pgc1a*, *Fgf21* and *Dio2* at 6 and 12 weeks of age in *Il18*^−/−^ mice was similar to the in vitro results (Figs. 1b–e, 2b, c). Thus, the in vivo results were similar to the in vitro results, suggesting that the histological and molecular differences were due to negative feedback of dyslipidemia.

To examine the molecular mechanism of lipid accumulation, comprehensive analysis was performed by microarray. Similar to our previous liver analyses, the function of ‘Quantity of adipose tissue’-related genes, which was common between 6 and 12 weeks of age, was extracted automatically, and *Apoc3*, *Insig1* and *Vdr* were detected (Fig. 3). *Apoc3* is closely associated with high density lipoprotein (HDL), modulates its function and induces BAT metabolic activation [52]. INSIG1 affects cholesterol biosynthesis [53]. In addition, increased expression of *Insig1* in the liver and in WAT may reduce cholesterol biosynthesis, resulting in obesity and dyslipidemia [54]. In our study, *Apoc3* and *Insig1* expression in *Il18*^−/−^ mice at 6 and 12 weeks of age was significantly increased compared with that in *Il18*^+/+^ mice (Fig. 3b). Thus, our results suggest that increased *Apoc3* expression may be induced by excessive lipid accumulation in BAT, and that increased *Insig1* was one of the mediators promoting fat accumulation.

As for *Vdr*, energy expenditure is increased in *VDR*-knockout mice, however, it is decreased in VDR-overexpressing mice [55, 56]. At 6 weeks of age, *Vdr* expression in *Il18*^−/−^ mice was increased, but at 12 weeks it significantly decreased (Fig. 3b). Therefore, our results suggest that at 12 weeks of age, decreased *Vdr* was a response to excessive lipid accumulation in BAT of *Il18*^−/−^ mice.

Although protein expression in BAs from *Il18*^−*/*−^ mice, which was significantly different such as UCP1 (Fig. 1), was not affected by rIL-18 administration (Additional file 6), in *Il18*^−/−^ mice, dyslipidemia was improved by short-term (2 weeks) rIL-18 administration [10]. Contrary to our expectation, the expression of PRDM16, FGF21 and PGC1α in BAT was similar. However, the increased expression of UCP1 in *Il18*^−/−^ mice was decreased by rIL-18 administration and there were no significant differences between the *Il18*^−/−^ and *Il18*^+/+^ mice (Fig. 4a, b). Regarding the other molecules, *Adrb3* and *Elovl3* were decreased and *Vdr* was increased after short-term rIL-18 treatment (Fig. 4c, d). Our results suggest that there was no direct effect of IL-18 on BAs, and lipid normalization by rIL-18 may result in decreased expression of *Adrb3*, *Elovl3*, *Vdr* and UCP1.

We have previously revealed that long-term intravenous administration of rIL-18 inhibited the body weight gain of *Il18*^+*/*+^ mice and prevented the onset of NASH in *Il18*^−/−^ mice [10]. Therefore, long-term exposure to excessive IL-18 expression may affect lipid homeostasis. Even though there was no effect on body weight in *Il18*^−/−^ mice, the expression of PGC1α and UCP1 in *Il18*^+*/*+^ mice after long-term rIL-18 treatment was significantly lower compared with that in mice treated with saline (Fig. 5a, b) [10]. Moreover, the size of cells containing lipids in BAT from *Il18*^−/−^ mice treated with rIL-18 from 37 to 49 weeks of age was much smaller than that of cells from *Il18*^+/+^ mice (Figs. 2a and 5c). Therefore, similar to previously shown liver results [10], IL-18 was indispensable for normal lipid metabolism not only in the liver, but also in BAT, and may contribute to the development of novel treatment options for lipid dyshomeostasis.

Limitations of the present study include a limited number of mice that received long-term (12 weeks) rIL-18 administration because of animal ethical restrictions enforced by the animal ethical committee in our college, which may be insufficient for obtaining statistical significance. Even though the direct effect on BAs under IL-18-deficient conditions in vitro was clarified, there were inconsistent results between the in vitro and in vivo experiments. Moreover, this study only focused on BAT. However, we are currently investigating the basic relationship between IL-18 and physiological homeostasis in other organs [10, 11]. Furthermore, additional analysis is required, for example, analysis of the metabolic function of IL-18 in BAT under cold exposure should be performed.

## Conclusions

We demonstrated novel roles of IL-18 in lipid metabolism in BAs and BAT. IL-18 deficiency led to increased expression of BA differentiation molecules, thus inducing BA differentiation and activation of the thermogenic function. In vivo, IL-18 impairment induced BAT to develop severe adiposity during growth. We also showed that molecules related to ‘Quantity of adipose tissue’, especially *Vdr*, were affected by IL-18 and may have a causal role in promoting lipid dyshomeostasis. In addition, rIL-18 administration improved both dyslipidemia and fat accumulation in BAT. Dyslipidemia can induce metabolic disorders such as obesity and lead to lethal diseases like cerebral infarction and myocardial infarction. IL-18 may have a causal role in maintaining the lipid concentration and may be a promising factor contributing to the development of novel treatment options for dyslipidemia through improving energy imbalance by lipids in BAT.

## Additional files

**Additional file 1.** Primer sequences used for qRT-PCR.

**Additional file 2.** Direct molecular pathway between IL-18, IL-18R and molecules related to differentiation and thermogenic functions. Direct molecular pathway of IL-18, IL-18R and genes related to differentiation and thermogenic functions in BAs. Based on previously published studies, there are no relationships between them.

**Additional file 3.** Heatmap analysis of gene expression profiles in BAT between *Il18*^+/+^ and *Il18*^−/−^ mice. Heatmap of microarray results at 6 (a) and 12 (b) weeks of age. The heatmap of three molecules identified in Fig. 3 at 6 (c) and 12 (d) weeks of age.

**Additional file 4.** Core analysis of molecules isolated at 6 weeks of age by microarray. Core analyses using IPA.

**Additional file 5.** Core analysis of molecules isolated at 12 weeks of age by microarray. Core analyses using IPA.

**Additional file 6.** Effect of rIL-18 on BAT precursor cells. Analysis of the effect of IL-18 on molecules shown in Fig. 1b and c. No difference was observed between the groups. BAT precursor cells extracted from *Il18*^−/−^ mice treated with saline (left) and rIL-18 (right). BAT: Brown adipose tissue; FABP4: Fatty acid-binding protein 4; FGF21: Fibroblast growth factor 21; PGC1α: Peroxisome proliferator-activated receptor γ coactivator 1-α; rIL-18: recombinant interleukin 18; PPARγ: Peroxisome proliferator-activated receptor γ; PRDM16: PR domain containing 16; UCP1: Uncoupling protein 1.

**Additional file 7.** IL-18 expression *in vitro*. IL-18 expression in each cell. Pre-BAs: BAT precursor cells; BAs: brown adipocyte; PC: positive control; rIL-18: recombinant interleukin 18.

## Abbreviations

*Adrb3*: adrenergic receptor beta 3
ANOVA: analysis of variance
*Apoc3*: apolipoprotein C-III
ATGL: adipose triglyceride lipase
Bas: brown adipocytes
BAT: brown adipose tissue
*Bmp7*: bone morphogenetic protein 7
*Cd36*: cluster of differentiation 36
*Cebpb*: CCAAT/enhancer binding protein (C/EBP) beta
*Cidea*: cell death activator CIDE-A
*Dio2*: type II iodothyronine deiodinase
*Elovl3*: elongation of very long chain fatty acids protein 3
FABP4: fatty acid-binding protein 4
*Fgf21*: fibroblast growth factor 21
GAPDH: glyceraldehyde-3-phosphate dehydrogenase
HDL: high density lipoprotein
HSL: hormone-sensitive lipase
IFN: interferon
*Insig1*: insulin-induced gene 1
IL-18: interleukin-18
*Il18*: interleukin-18
*Il18*^−/−^: IL-18 knockout
IPA: Ingenuity^®^ Pathway Analysis
*Lep*: leptin
*Myf5*: *myogenic factor 5*
NAFLD: non-alcoholic fatty liver disease
NASH: non-alcoholic steatohepatitis
PBS: phosphate-buffered saline
*Pgc1a*: peroxisome proliferator-activated receptor γ coactivator 1-α
*Pgc1b*: peroxisome proliferator-activated receptor γ coactivator 1-β
PPARγ, *Pparγ*: peroxisome proliferator-activated receptor γ
PRDM16: PR domain containing 16
rIL-18: recombinant IL-18
qRT-PCR: quantitative reverse transcription polymerase chain reaction
T3: triiodothyronine
TPBS: PBS containing 0.1% Triton X-100
*Ucp1*: uncoupling protein-1
*Ucp3*: uncoupling protein 3
*Vdr*: vitamin D (1,25- dihydroxyvitamin D3) receptor
WAT: white adipose tissue

## References

[1] Marchesini G, Bugianesi E, Forlani G, Cerrelli F, Lenzi M, Manini R, Natale S, Vanni E, Villanova N, Melchionda N, Rizzetto M (2003). Nonalcoholic fatty liver, steatohepatitis, and the metabolic syndrome. Hepatology.

[2] Gami AS, Witt BJ, Howard DE, Erwin PJ, Gami LA, Somers VK, Montori VM (2007). Metabolic syndrome and risk of incident cardiovascular events and death: a systematic review and meta-analysis of longitudinal studies. J Am Coll Cardiol.

[3] Okamura H, Tsutsi H, Komatsu T, Yutsudo M, Hakura A, Tanimoto T, Torigoe K, Okura T, Nukada Y, Hattori K (1995). Cloning of a new cytokine that induces IFN-gamma production by T cells. Nature.

[4] Reddy P (2004). Interleukin-18: recent advances. Curr Opin Hematol.

[5] Ghayur T, Banerjee S, Hugunin M, Butler D, Herzog L, Carter A, Quintal L, Sekut L, Talanian R, Paskind M (1997). Caspase-1 processes IFN-gamma-inducing factor and regulates LPS-induced IFN-gamma production. Nature.

[6] Okamura H, Tsutsui H, Kashiwamura S, Yoshimoto T, Nakanishi K (1998). Interleukin-18: a novel cytokine that augments both innate and acquired immunity. Adv Immunol.

[7] Sugawara S, Uehara A, Nochi T, Yamaguchi T, Ueda H, Sugiyama A, Hanzawa K, Kumagai K, Okamura H, Takada H (2001). Neutrophil proteinase 3-mediated induction of bioactive IL-18 secretion by human oral epithelial cells. J Immunol.

[8] Tsutsui H, Kayagaki N, Kuida K, Nakano H, Hayashi N, Takeda K, Matsui K, Kashiwamura S, Hada T, Akira S (1999). Caspase-1-independent, Fas/Fas ligand-mediated IL-18 secretion from macrophages causes acute liver injury in mice. Immunity.

[9] Netea MG, Joosten LA, Lewis E, Jensen DR, Voshol PJ, Kullberg BJ, Tack CJ, van Krieken H, Kim SH, Stalenhoef AF (2006). Deficiency of interleukin-18 in mice leads to hyperphagia, obesity and insulin resistance. Nat Med.

[10] Yamanishi K, Maeda S, Kuwahara-Otani S, Watanabe Y, Yoshida M, Ikubo K, Okuzaki D, El-Darawish Y, Li W, Nakasho K (2016). Interleukin-18-deficient mice develop dyslipidemia resulting in nonalcoholic fatty liver disease and steatohepatitis. Transl Res.

[11] Yamanishi K, Mukai K, Hashimoto T, Ikubo K, Nakasho K, El-Darawish Y, Li W, Okuzaki D, Watanabe Y, Hayakawa T (2018). Physiological and molecular effects of interleukin-18 administration on the mouse kidney. J Transl Med.

[12] Moriwaki Y, Yamamoto T, Shibutani Y, Aoki E, Tsutsumi Z, Takahashi S, Okamura H, Koga M, Fukuchi M, Hada T (2003). Elevated levels of interleukin-18 and tumor necrosis factor-alpha in serum of patients with type 2 diabetes mellitus: relationship with diabetic nephropathy. Metabolism.

[13] Yamaoka-Tojo M, Tojo T, Wakaume K, Kameda R, Nemoto S, Takahira N, Masuda T, Izumi T (2011). Circulating interleukin-18: a specific biomarker for atherosclerosis-prone patients with metabolic syndrome. Nutr Metab (Lond).

[14] Seale P, Bjork B, Yang W, Kajimura S, Chin S, Kuang S, Scime A, Devarakonda S, Conroe HM, Erdjument-Bromage H (2008). PRDM16 controls a brown fat/skeletal muscle switch. Nature.

[15] Kajimura S, Seale P, Kubota K, Lunsford E, Frangioni JV, Gygi SP, Spiegelman BM (2009). Initiation of myoblast to brown fat switch by a PRDM16-C/EBP-beta transcriptional complex. Nature.

[16] Seale P, Kajimura S, Yang W, Chin S, Rohas LM, Uldry M, Tavernier G, Langin D, Spiegelman BM (2007). Transcriptional control of brown fat determination by PRDM16. Cell Metab.

[17] Aquila H, Link TA, Klingenberg M (1985). The uncoupling protein from brown fat mitochondria is related to the mitochondrial ADP/ATP carrier. Analysis of sequence homologies and of folding of the protein in the membrane. EMBO J.

[18] Bouillaud F, Weissenbach J, Ricquier D (1986). Complete cDNA-derived amino acid sequence of rat brown fat uncoupling protein. J Biol Chem.

[19] Heaton GM, Wagenvoord RJ, Kemp A, Nicholls DG (1978). Brown-adipose-tissue mitochondria: photoaffinity labelling of the regulatory site of energy dissipation. Eur J Biochem.

[20] Ridley RG, Patel HV, Gerber GE, Morton RC, Freeman KB (1986). Complete nucleotide and derived amino acid sequence of cDNA encoding the mitochondrial uncoupling protein of rat brown adipose tissue: lack of a mitochondrial targeting presequence. Nucleic Acids Res.

[21] Cannon B, Nedergaard J (2004). Brown adipose tissue: function and physiological significance. Physiol Rev.

[22] Enerback S, Jacobsson A, Simpson EM, Guerra C, Yamashita H, Harper ME, Kozak LP (1997). Mice lacking mitochondrial uncoupling protein are cold-sensitive but not obese. Nature.

[23] Orava J, Nuutila P, Lidell ME, Oikonen V, Noponen T, Viljanen T, Scheinin M, Taittonen M, Niemi T, Enerback S, Virtanen KA (2011). Different metabolic responses of human brown adipose tissue to activation by cold and insulin. Cell Metab.

[24] Virtanen KA, Lidell ME, Orava J, Heglind M, Westergren R, Niemi T, Taittonen M, Laine J, Savisto NJ, Enerback S, Nuutila P (2009). Functional brown adipose tissue in healthy adults. N Engl J Med.

[25] van Marken Lichtenbelt WD, Vanhommerig JW, Smulders NM, Drossaerts JM, Kemerink GJ, Bouvy ND, Schrauwen P, Teule GJ (2009). Cold-activated brown adipose tissue in healthy men. N Engl J Med.

[26] Lowell BB, Vedrana SS, Hamann A, Lawitts JA, Himms-Hagen J, Boyer BB, Kozak LP, Flier JS (1993). Development of obesity in transgenic mice after genetic ablation of brown adipose tissue. Nature.

[27] Takeda K, Tsutsui H, Yoshimoto T, Adachi O, Yoshida N, Kishimoto T, Okamura H, Nakanishi K, Akira S (1998). Defective NK cell activity and Th1 response in IL-18-deficient mice. Immunity.

[28] Yamanishi K, Doe N, Sumida M, Watanabe Y, Yoshida M, Yamamoto H, Xu Y, Li W, Yamanishi H, Okamura H, Matsunaga H (2015). Hepatocyte nuclear factor 4 alpha is a key factor related to depression and physiological homeostasis in the mouse brain. PLoS ONE.

[29] Metsalu T, Vilo J (2015). ClustVis: a web tool for visualizing clustering of multivariate data using Principal Component Analysis and heatmap. Nucleic Acids Res.

[30] Farmer SR (2006). Transcriptional control of adipocyte formation. Cell Metab.

[31] Kajimura S, Seale P, Spiegelman BM (2010). Transcriptional control of brown fat development. Cell Metab.

[32] Puigserver P, Wu Z, Park CW, Graves R, Wright M, Spiegelman BM (1998). A cold-inducible coactivator of nuclear receptors linked to adaptive thermogenesis. Cell.

[33] Klingenberg M (1999). Uncoupling protein—a useful energy dissipator. J Bioenerg Biomembr.

[34] Kajimura S, Seale P, Tomaru T, Erdjument-Bromage H, Cooper MP, Ruas JL, Chin S, Tempst P, Lazar MA, Spiegelman BM (2008). Regulation of the brown and white fat gene programs through a PRDM16/CtBP transcriptional complex. Genes Dev.

[35] Badman MK, Pissios P, Kennedy AR, Koukos G, Flier JS, Maratos-Flier E (2007). Hepatic fibroblast growth factor 21 is regulated by PPARalpha and is a key mediator of hepatic lipid metabolism in ketotic states. Cell Metab.

[36] Kharitonenkov A, Shiyanova TL, Koester A, Ford AM, Micanovic R, Galbreath EJ, Sandusky GE, Hammond LJ, Moyers JS, Owens RA (2005). FGF-21 as a novel metabolic regulator. J Clin Invest.

[37] Hondares E, Rosell M, Gonzalez FJ, Giralt M, Iglesias R, Villarroya F (2010). Hepatic FGF21 expression is induced at birth via PPARalpha in response to milk intake and contributes to thermogenic activation of neonatal brown fat. Cell Metab.

[38] Hondares E, Iglesias R, Giralt A, Gonzalez FJ, Giralt M, Mampel T, Villarroya F (2011). Thermogenic activation induces FGF21 expression and release in brown adipose tissue. J Biol Chem.

[39] Furuhashi M, Tuncman G, Gorgun CZ, Makowski L, Atsumi G, Vaillancourt E, Kono K, Babaev VR, Fazio S, Linton MF (2007). Treatment of diabetes and atherosclerosis by inhibiting fatty-acid-binding protein aP2. Nature.

[40] Furuhashi M, Hotamisligil GS (2008). Fatty acid-binding proteins: role in metabolic diseases and potential as drug targets. Nat Rev Drug Discov.

[41] Furuhashi M, Ishimura S, Ota H, Miura T (2011). Lipid chaperones and metabolic inflammation. Int J Inflamm.

[42] Tontonoz P, Graves RA, Budavari AI, Erdjument-Bromage H, Lui M, Hu E, Tempst P, Spiegelman BM (1994). Adipocyte-specific transcription factor ARF6 is a heterodimeric complex of two nuclear hormone receptors, PPAR gamma and RXR alpha. Nucleic Acids Res.

[43] Adida A, Spener F (2006). Adipocyte-type fatty acid-binding protein as inter-compartmental shuttle for peroxisome proliferator activated receptor gamma agonists in cultured cell. Biochim Biophys Acta.

[44] Ahmadian M, Duncan RE, Varady KA, Frasson D, Hellerstein MK, Birkenfeld AL, Samuel VT, Shulman GI, Wang Y, Kang C, Sul HS (2009). Adipose overexpression of desnutrin promotes fatty acid use and attenuates diet-induced obesity. Diabetes.

[45] Ahmadian M, Abbott MJ, Tang T, Hudak CS, Kim Y, Bruss M, Hellerstein MK, Lee HY, Samuel VT, Shulman GI (2011). Desnutrin/ATGL is regulated by AMPK and is required for a brown adipose phenotype. Cell Metab.

[46] Degerman E, Smith CJ, Tornqvist H, Vasta V, Belfrage P, Manganiello VC (1990). Evidence that insulin and isoprenaline activate the cGMP-inhibited low-Km cAMP phosphodiesterase in rat fat cells by phosphorylation. Proc Natl Acad Sci USA.

[47] Anthonsen MW, Ronnstrand L, Wernstedt C, Degerman E, Holm C (1998). Identification of novel phosphorylation sites in hormone-sensitive lipase that are phosphorylated in response to isoproterenol and govern activation properties in vitro. J Biol Chem.

[48] Garton AJ, Yeaman SJ (1990). Identification and role of the basal phosphorylation site on hormone-sensitive lipase. Eur J Biochem.

[49] Cypess AM, Weiner LS, Roberts-Toler C, Franquet Elia E, Kessler SH, Kahn PA, English J, Chatman K, Trauger SA, Doria A, Kolodny GM (2015). Activation of human brown adipose tissue by a beta3-adrenergic receptor agonist. Cell Metab.

[50] de Jesus LA, Carvalho SD, Ribeiro MO, Schneider M, Kim SW, Harney JW, Larsen PR, Bianco AC (2001). The type 2 iodothyronine deiodinase is essential for adaptive thermogenesis in brown adipose tissue. J Clin Invest.

[51] Pellegrino MJ, McCully BH, Habecker BA (2014). Leptin stimulates sympathetic axon outgrowth. Neurosci Lett.

[52] Zvintzou E, Lhomme M, Chasapi S, Filou S, Theodoropoulos V, Xapapadaki E, Kontush A, Spyroulias G, Tellis CC, Tselepis AD (2017). Pleiotropic effects of apolipoprotein C3 on HDL functionality and adipose tissue metabolic activity. J Lipid Res.

[53] Dong XY, Tang SQ (2010). Insulin-induced gene: a new regulator in lipid metabolism. Peptides.

[54] Decara JM, Romero-Cuevas M, Rivera P, Macias-Gonzalez M, Vida M, Pavon FJ, Serrano A, Cano C, Fresno N, Perez-Fernandez R (2012). Elaidyl-sulfamide, an oleoylethanolamide-modelled PPARalpha agonist, reduces body weight gain and plasma cholesterol in rats. Dis Model Mech.

[55] Wong KE, Szeto FL, Zhang W, Ye H, Kong J, Zhang Z, Sun XJ, Li YC (2009). Involvement of the vitamin D receptor in energy metabolism: regulation of uncoupling proteins. Am J Physiol Endocrinol Metab.

[56] Wong KE, Kong J, Zhang W, Szeto FL, Ye H, Deb DK, Brady MJ, Li YC (2011). Targeted expression of human vitamin D receptor in adipocytes decreases energy expenditure and induces obesity in mice. J Biol Chem.

