# Supplementary figures and images for: Deficiency in interleukin-18 promotes differentiation of brown adipose tissue resulting in fat accumulation despite dyslipidemia
Source: J Transl Med. 2018 Nov 19;16:314. doi: 10.1186/s12967-018-1684-3 (PMC6245626; doi:10.1186/s12967-018-1684-3)

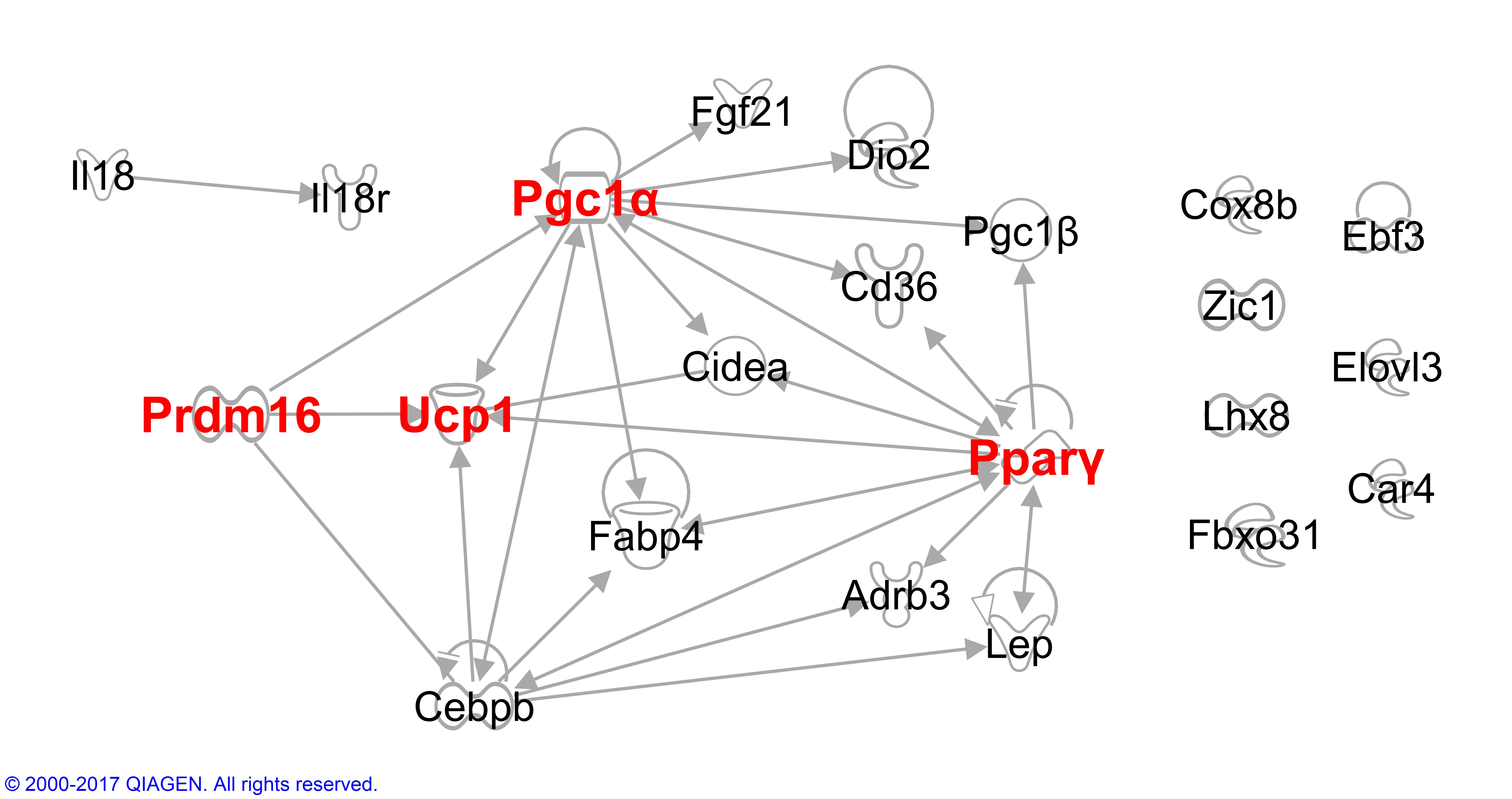

Supplement: Supplementary file 2 — Additional file 2. Direct molecular pathway between IL-18, IL-18R and molecules related to differentiation and thermogenic functions. Direct molecular pathway of IL-18, IL-18R and genes related to differentiation and thermogenic functions in BAs. Based on previously published studies, there are no relationships between them. [file 12967_2018_1684_MOESM2_ESM.jpg]

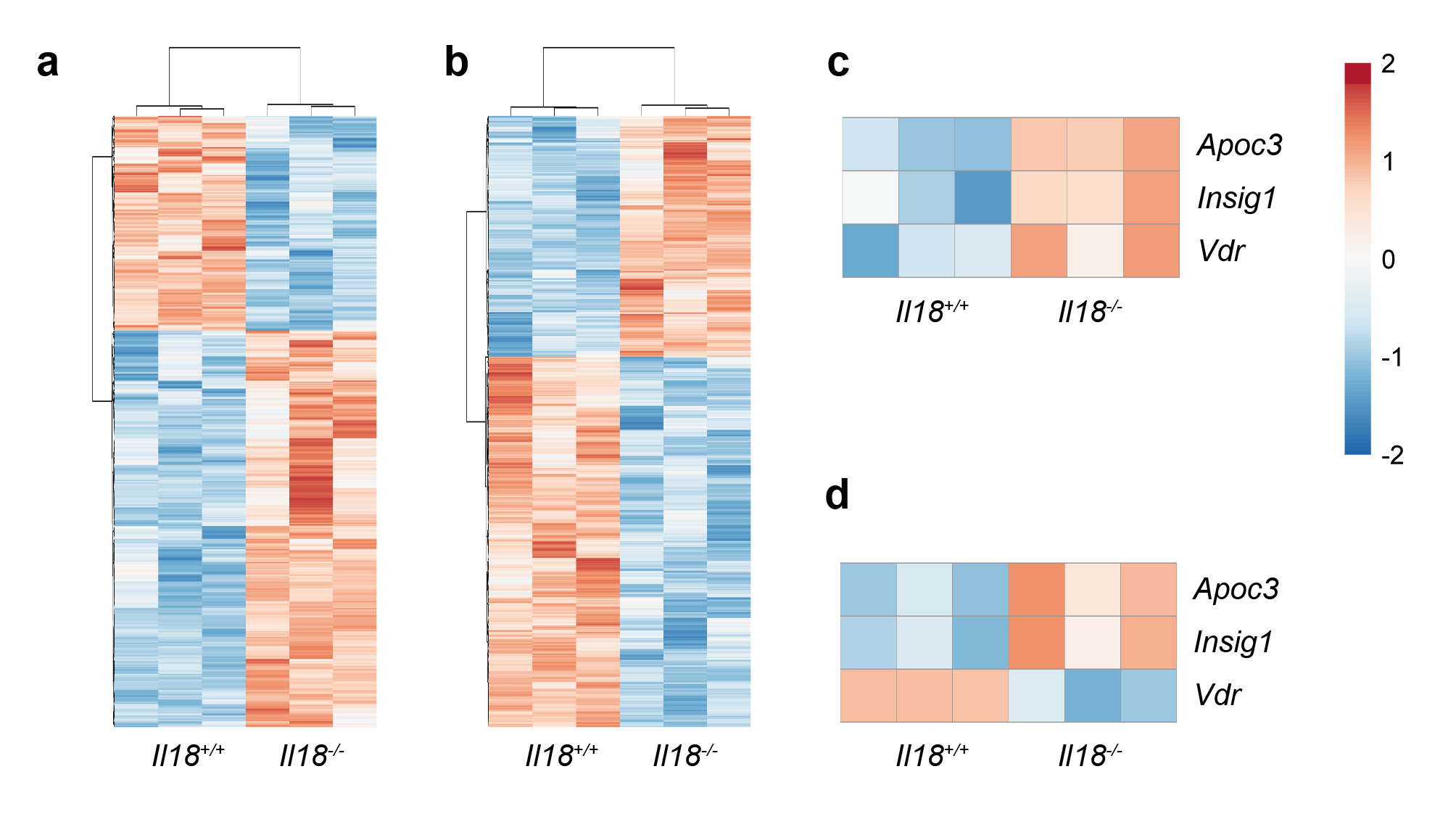

Supplement: Supplementary file 3 — Additional file 3. Heatmap analysis of gene expression profiles in BAT between Il18+/+ and Il18−/− mice. Heatmap of microarray results at 6 (a) and 12 (b) weeks of age. The heatmap of three molecules identified in Fig. 3 at 6 (c) and 12 (d) weeks of age. [file 12967_2018_1684_MOESM3_ESM.tif]

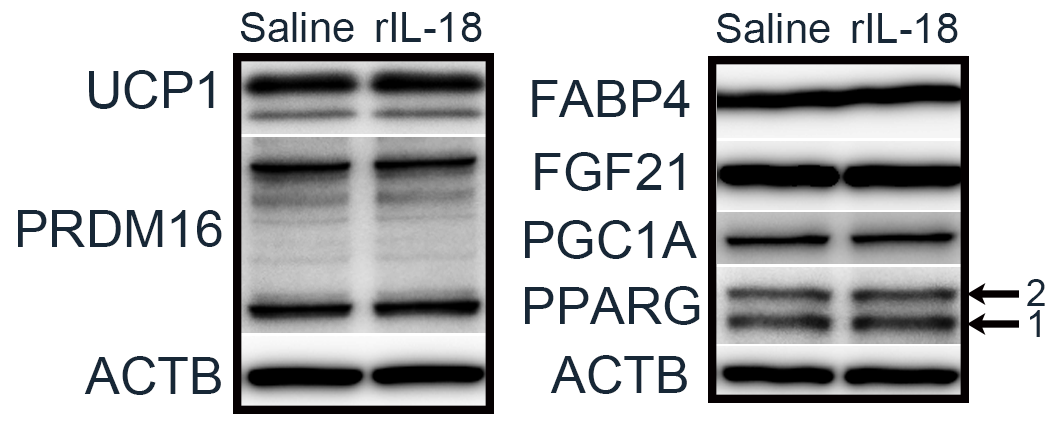

Supplement: Supplementary file 6 — Additional file 6. Effect of rIL-18 on BAT precursor cells. Analysis of the effect of IL-18 on molecules shown in Fig. 1b and c. No difference was observed between the groups. BAT precursor cells extracted from Il18−/− mice treated with saline (left) and rIL-18 (right). BAT: Brown adipose tissue; FABP4: Fatty acid-binding protein 4; FGF21: Fibroblast growth factor 21; PGC1α: Peroxisome proliferator-activated receptor γ coactivator 1-α; rIL-18: recombinant interleukin 18; PPARγ: Peroxisome proliferator-activated receptor γ; PRDM16: PR domain containing 16; UCP1: Uncoupling protein 1. [file 12967_2018_1684_MOESM6_ESM.tif]

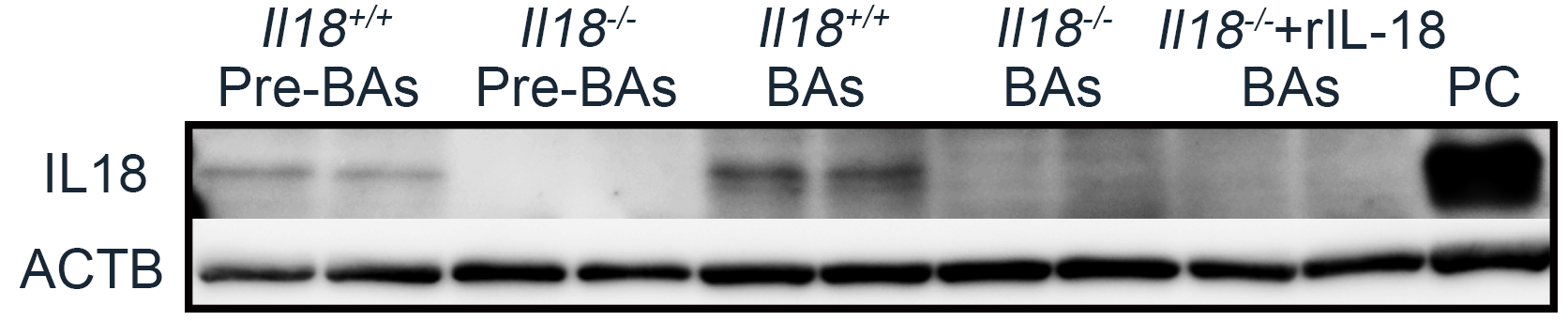

Supplement: Supplementary file 7 — Additional file 7. IL-18 expression in vitro. IL-18 expression in each cell. Pre-BAs: BAT precursor cells; BAs: brown adipocyte; PC: positive control; rIL-18: recombinant interleukin 18. [file 12967_2018_1684_MOESM7_ESM.tif]
